# Endoscopic Band Ligation Is Able to Close Perforations Caused by Colonoscopy: A Porcine Model Study

**DOI:** 10.1155/2018/4325675

**Published:** 2018-04-01

**Authors:** Yidong Yang, Xianyi Lin, Siwei Tan, Xiaoli Huang, Zijun Xie, Xuan Xu, Yiming Lei, Bin Wu

**Affiliations:** Department of Gastroenterology and Endoscopy, The Third Affiliated Hospital of Sun Yat-sen University, Guangzhou, China

## Abstract

**Objective:**

Diagnostic colonoscopy is important for diagnosing colorectal diseases, including inflammatory bowel disease and colorectal tumours. Perforation during diagnostic colonoscopy, a rare but serious complication, is a considerable factor before performing the procedure. Immediate endoluminal closure of a perforation could prevent the adverse consequences associated with general anaesthesia and surgery. This study is aimed at assessing the potential effectiveness and safety of endoscopic band ligation (EBL) in closing a colon perforation during endoscopy in a porcine model.

**Methods:**

Colon perforations were created and then subsequently closed with EBL in six porcine models. After 28 days of careful follow-up, pigs were euthanized for clinical and pathologic evaluations.

**Results:**

All colon perforations were successfully closed using EBL in pigs. The mean time of perforation closure with EBL was 244.3 seconds with one to two bands, and there were no immediate complications or clinical manifestations of peritonitis or sepsis in any animals. No pericolonic abscess or peritonitis was found during necropsy. Histopathology demonstrated reepithelialization of the mucosa at the perforation site.

**Conclusions:**

Immediate closure of perforations caused during colonoscopy with EBL is feasible and safe in a porcine model.

## 1. Introduction

Diagnostic colonoscopy is considered to be a first choice tool for diagnosing colorectal diseases in many countries worldwide, as supported by the accumulated evidence of the efficacy of diagnosing colorectal diseases early, leading to reduced mortality, especially for colorectal cancer [1]. However, iatrogenic colonic perforation during diagnostic colonoscopy is a potentially life-threatening and devastating adverse event that requires emergency management and can cause considerable unexpected hospitalizations and even death in healthy people [2].

Recently, a variety of techniques have been reported for endoluminal closing of perforations during colonoscopy. It is recommended that through-the-scope (TTS) clips be used for endoluminal closure of small perforations. However, perforation size (<10 mm) limitations as well as tangential angles or wide gaps formed in the anatomic site of the perforation restrict the usage of TTS [3–6]. Although over-the-scope clips (OTSCs) are recommended for larger colonic perforations (>10 mm) as the first choice in specific cases, their high cost limits their utility in developing countries. A few novel devices have been developed to overcome the limitations of endoclipping, although the majority remain as prototypes or are not readily available in many countries [7]. Endoscopic band ligation (EBL) is a first-line treatment for gastroesophageal varices or variceal bleeding in cirrhosis patients. Recently, gastrointestinal perforations, including perforations formed in the stomach, duodenum, and colon, have been closed by EBL, with a very high success rate *in vivo* and in some clinical cases [8, 9].

The purpose of our study was to assess the feasibility and safety of colonic perforation closure by using EBL. We created colon perforations in a porcine model *in vivo*. Perforations were closed with EBL. Signs of peritonitis or sepsis in animals were inspected for 4 weeks. Pigs were euthanized at 2 or 4 weeks to study the endoscopic closure of colonic perforations with EBL.

## 2. Materials and Methods

This research was approved by the Sun Yat-sen University Institutional Animal Care and Use Committee and conducted in a dedicated animal facility of Olympus Company (Guangzhou, China). Six adult female swine weighing 23 to 29.5 kg were used. After the EBL closure operation for colonic perforations, animals were observed daily for clinical signs of peritonitis and sepsis, and colon endoscopies were performed on days 7, 14, and 28. Pigs were killed 2 or 4 weeks after EBL closure to assess peritonitis, wound healing, and the colon pathology.

### 2.1. Preparation before Colonoscopy and the Anaesthesia Procedure

Before laxatives were provided for bowel preparation, animals consumed a liquid diet for 3 days. One day before colonoscopy, the colon was cleaned with 500 ml of a polyethylene glycol electrolyte solution (Wanhe Pharmacy Co. Ltd, Shenzhen, China) two times. The colonoscopy was performed with the animals in the left lateral position. The sedative consisted of xylazine (2 mg/kg), and ketamine (2 mg/kg) was given as the preanaesthesia. After endotracheal intubation, general inhalation anaesthesia was maintained by using 1% isoflurane and a N_2_O/O_2_ mixture during colonoscopy.

### 2.2. Creation and Endoscopic Closure of Perforations

**Figure 1:**
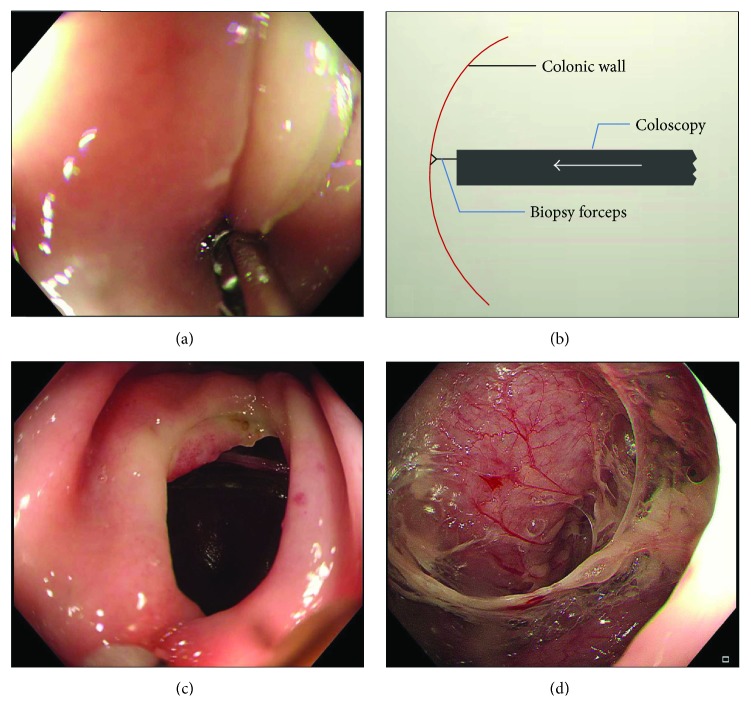
Process of colonic perforation creation. (a) A biopsy forceps with needle is first clamped on the colonic wall to fix the coloscope during the creation of the perforation. (b) The schematic plan shows that the coloscope is pushed toward to the colon wall vertically to perforate the colonic wall and create a perforation. (c) A 1.2–1.5 cm full-thickness perforation of the colon is made. (d) The size and full-thickness nature of the perforation are verified by passing the endoscope into the free abdominal cavity.

**Figure 2:**
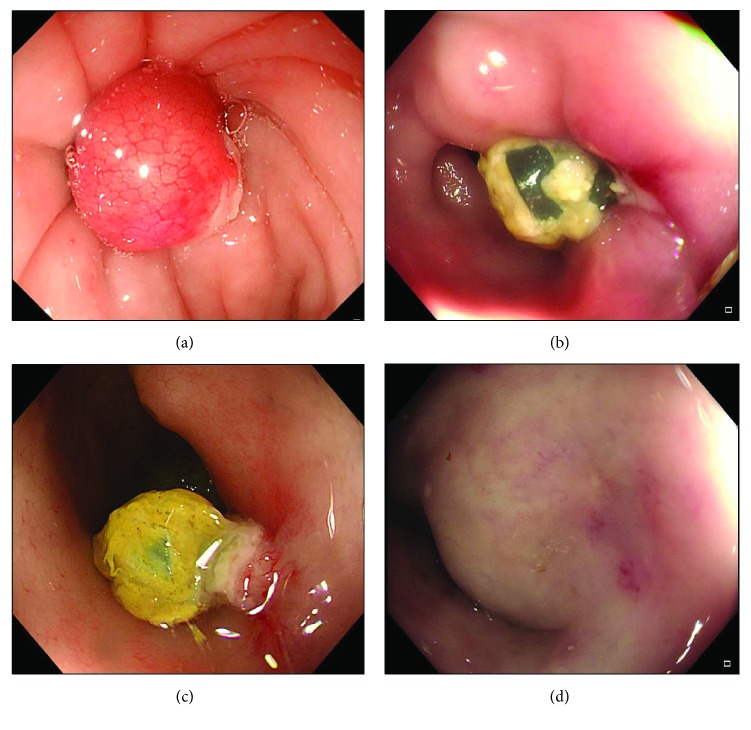
Follow-up of pigs after the operation with coloscopy. (a) Endoscopic band ligation is used to close the perforation. (b) Follow-up colon endoscopy performed on day 7 showing a white coating with band. (c) On day 14, endoscopy revealed a white coating with band. (d) An internal view of the perforation site by endoscopy shows a completely healed scar.

A colon endoscope (CF-HQ290I, Olympus Optical Co. Ltd., Tokyo, Japan) was used. A biopsy forceps with a needle was used to assist in generating full-thickness longitudinal colonic perforations located 15 to 20 cm from the anus in each pig. During the creation of the perforation, the biopsy forceps with a needle was first clamped on the colonic wall to fix the coloscope (Figure 1(a)). Then, the coloscope was pushed toward to the colon wall vertically to perforate the colonic wall and create a perforation (Figures 1(b) and 1(c)). The size and full-thickness nature of each perforation were verified by pushing the coloscope tip into the free abdominal cavity during each operation (Figure 1(d)). The size of each perforation was approximately 1.2–1.5 cm. Further details can be observed in the Supplementary video (available here). Endoscopic closure of the colon perforation was performed with a gastric endoscope (GIF-HQ260J, Olympus Optical Co. Ltd., Tokyo, Japan) and a 6-Shooter Universal Saeed Multi-Band Ligator (Cook Medical, Bloomington, IN, USA ) following the manufacturer's procedures. Whole perforated colonic tissue was gently aspirated into the barrel, maintaining suction and deploying the band. If the perforation was closed incompletely after the first ligation, a second EBL was performed to completely close the perforation (Figure 2(a)).

### 2.3. Follow-Up

All pigs were fasted for the first 24 hours after recovery from the operation. A liquid diet was given for the next 24 hours, and then, a regular diet was given. All animals were given ciprofloxacin (15 mg/kg) orally twice per day for 3 days after the operation. General symptoms and signs of sepsis or peritonitis of the animals were closely monitored.

### 2.4. Necropsy

Animals were subsequently killed for clinical and pathologic evaluations after 2 or 4 weeks. Laparotomy through a midline incision was performed to the peritoneal cavity. The abdominal cavity was closely inspected for adhesions or abscesses. The segment of the colon with injury related to closure of the perforation was identified, isolated, and evaluated for healing of the mucosa. Then, colon tissues with perforation closure were examined for histopathological healing.

The following six parameters were studied to assess the outcomes of perforation closure with EBL: (1) technical feasibility of the procedure; (2) duration of each perforation closure; (3) colon endoscopy results on days 7, 14, and 28; (4) clinical monitoring of the animals for peritonitis and sepsis; (5) necropsy at 2 or 4 weeks to check for peritonitis and sepsis; and (6) evaluation of histologic healing.

## 3. Results

### 3.1. Technical Feasibility and Clinical Outcomes

Closure of colon perforations with EBL was performed successfully in six animals (100%) within a mean time of 244.3 seconds. The mean quantity of the bands used was 1.5 (Table 1). Incomplete suction of perforated lesion tissue resulted in a prolonged procedure time. After EBL, complete closure of the colon perforation was confirmed by full distension with air insufflation. No immediate procedure-related organ injuries or bleeding were encountered. A follow-up colon endoscopy performed on days 7 and 14 showed a white coating with a band (Figures 2(b) and 2(c)) and on day 28 revealed complete healing of the perforation (Figure 2(d)). All six animals survived without complications.

### 3.2. Necropsy Findings

**Figure 3:**
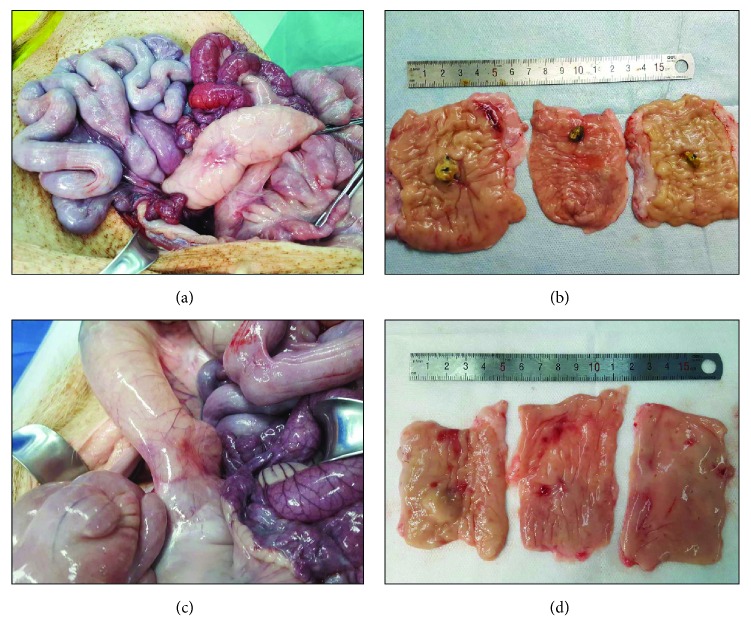
Results of necropsy. (a) An external view of the perforation site shows no adhesion with other organs on days 14. (b) An internal view of the perforation site shows a white coating with a band. (c) On days 28, a macroscopic view of the external of the perforation site shows no adhesion. (d) Internal view of the perforation sites shows completely healed scars.

**Figure 4:**
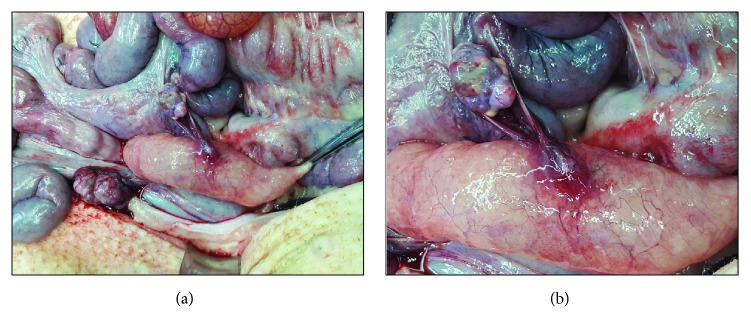
Macroscopic view of local adhesion. (a) An external macroscopic view of the perforation site shows local adhesion with a fibrous band and distant adhesion with the small bowel in one case. (b) Magnified view of the adhesion in the perforation site.

No transmural wound dehiscence, peritonitis, or pericolic abscesses were found on the necropsy examination of the animals. The perforation site after 2 weeks had a white coating with band (Figures 3(a) and 3(b)). After 4 weeks, the injury site was completely covered with a scar and regenerated epithelium (Figures 3(c) and 3(d)). In one case, peritoneum suction during EBL resulted in local adhesion of the colon and small intestine with a thin fibrous band without bowel obstruction (Figure 4).

### 3.3. Histopathological Examination

**Figure 5:**
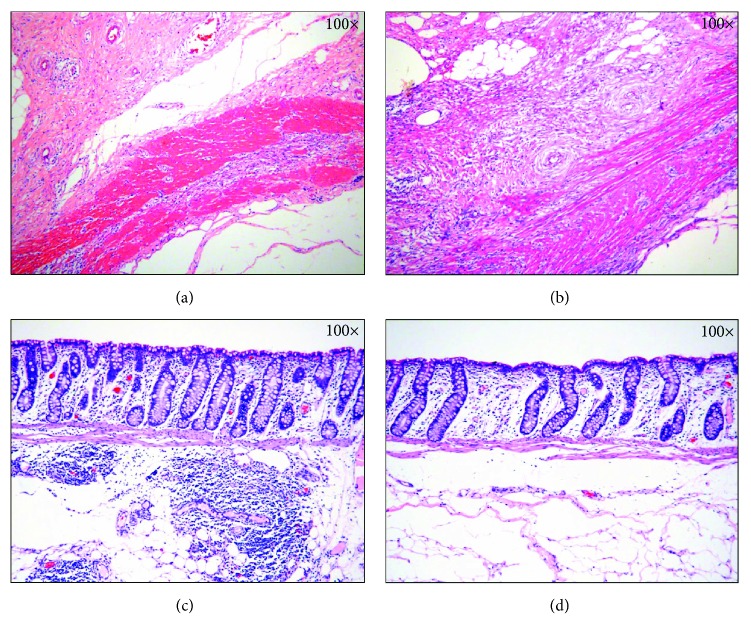
Histologic results of the perforation site after EBL. (a) Moderate fibrosis with chronic inflammation is observed in the submucosa (H&E stain, ×100). (b) Fibrotic tissue replaced the defect of the submucosa and muscularis propria (H&E stain, ×100). (c) Infiltration of inflammatory cells and granulation tissue in the submucosa is observed. (H&E stain, ×100). (d) Healing with reepithelialization of the mucosa (H&E stain, ×100).

The histopathological results showed that the perforation sites were completely closed in all pigs. The surface of the healing site was majorly covered with a fibrotic scar and partly covered with mucosal reepithelialization. Inflamed granulation tissues were found in the submucosa layer, and fibrotic tissue replaced the defect of the submucosa and muscularis propria (Figure 5).

## 4. Discussion

Perforation during diagnostic colonoscopy is an uncommon complication but is associated with a relatively high mortality rate [10]. Reports have shown that the incidence rate of perforation is 0.03–0.8% during diagnostic colonoscopy [11]. Surgical closure through laparotomy or laparoscopy is an effective management for perforation, although there is a risk of complications, such as anaesthetic accident, infection, and ileus [12]. Successful mini-invasive management of colon perforations by endoluminal repair via TTS clips or OTSCs could reduce potential risks of surgical closure [13]. However, TTS clips require enough space be available to pass the scope under direct visualization and might not successfully repair large perforations (>10 mm). However, OTSCs or some novel advanced devices might not be readily available in many countries, especially in developing countries due to economic reasons.

EBL is an effective, safe, and widely used treatment for gastroesophageal varices; bleeding of cirrhosis; vessel disorders related to bleeding, such as Dieulafoy's ulcer or gastric angiodysplasia; and excision of small gastrointestinal stromal tumours [14]. According to recent studies, EBL has been reported to be a salvage option for closing perforations of the stomach, duodenum, colon, or rectum after endoclip failure. A case report containing 5 patients evaluated the feasibility of EBL as an alternative choice for gastric perforation repair after failure to close with endoclips [11]. Similar studies on closure with EBL as a salvage technique for colon perforations and rectal perforation caused by ESD after endoclip failure have been reported [15–17]. Closure of the perforations in all cases was successful. In an *in vivo* canine model, EBL was reported to be a feasible technique for closure of iatrogenic colon perforations caused by needle knives [8].

In this *in vivo* study, we used a method that simulated iatrogenic colonic perforations during diagnostic colonoscopy to form 1.2–1.5 cm colon perforations in porcine models. During the repair of perforations in six pigs, we found that EBL was easy and safe for performing for colonic perforation closure and was without complications. The results from the follow-up and necropsy of the animals after closure showed complete closure of perforation in all animals without peritonitis or sepsis. Histopathological examination by H&E reflected total healing of the injury site. All of the above results indicate the technical feasibility of EBL for the closure of colonic perforations that are not larger than 1.5 cm and suggest that EBL could be considered to be a salvage method for iatrogenic perforations that form during coloscopy after endoclip failure.

Every novel developing technique or device has its limitations [18]. EBL might aspirate more tissue than needed or serous membrane and cause injury to adjacent organs during the suctioning of the perforation site. The consequence of serous membrane involvement in the closure of colon perforation is controversial. Studies have demonstrated that the involvement of serous membrane during the repair of large perforations might increase blood supply and improve the quality of closure by accelerating the healing speed of injured tissue. However, some studies have indicated that serous membrane involvement might increase the risk of bacterial infection and cause peritonitis or sepsis [8, 17, 19]. In our study, it was not easy to avoid aspiration of the serous membrane, even though EBL was performed carefully. The serous membrane was drawn in during aspiration in three cases, and one case showed adhesion to another organ. However, our cases showed no clinical problems. Therefore, aspiration of the serous membrane during EBL might be helpful for perforation closure and patient prognosis. In our study, bands on the perforation site were still be found at day 14, while Han et al. showed ulcer or scar formation without bands on the perforation [8]. This difference might have results from the different species used for the study, and actual outcomes in humans require further investigation. The other limitation of EBL is that the endoscope needs to be withdrawn to install the band ligation device before perforation closure, especially for perforations located at the distal colon. However, the majority of iatrogenic perforations occur in the sigmoid colon or the rectosigmoid junction [2], locations that are not difficult to reach during endoscopy with the band ligation device.

This study reveals that EBL might be reliable for closing colon perforations. The EBL device is easy to use, and the technique is easy to perform safely to reach ideal outcomes. The involvement of the serous membrane in the closure occurred in our study without disadvantageous consequences. Further investigations, including a larger sample size and comparative survival study, are needed to evaluate the reliability and safety of EBL to expand its clinical use.

## Figures and Tables

**Table 1:** Technical results and clinical outcomes in a colon perforation closure model.

Number	Quantity of bands for perforation closure	Duration of closure (second)	Aspiration of serous membrane into the suction cup	Peritonitis	Survival situation
1	1	192	Yes	No	Survival for 2 weeks
2	2	251	No	No	Survival for 2 weeks
3	2	314	Yes	No	Survival for 2 weeks
4	1	227	No	No	Survival for 4 weeks
5	1	176	No	No	Survival for 4 weeks
6	2	306	Yes	No	Survival for 4 weeks
Mean ± SD	1.5 ± 0.5	244.3 ± 57.3			

## Abbreviations

EBL: Endoscopic band ligation
TTS: Through-the-scope
OTSCs: Over-the-scope clips.

## Supplementary Materials

Supplementary Materials Supplementary video: process of an iatrogenic colon perforation model, perforation closure, and follow-up colon endoscopy on days 7, 14, and 28.
